# Quantitative proteomics unravels that the post-transcriptional regulator Crc modulates the generation of vesicles and secreted virulence determinants of *Pseudomonas aeruginosa*

**DOI:** 10.1016/j.dib.2015.07.002

**Published:** 2015-07-10

**Authors:** Jose Antonio Reales-Calderón, Fernando Corona, Lucía Monteoliva, Concha Gil, Jose Luis Martínez

**Affiliations:** a Departamento de Biotecnología Microbiana, Centro Nacional de Biotecnología, CSIC, Madrid, Spain; b Departamento de Microbiología II, Facultad de Farmacia, Universidad Complutense de Madrid and Instituto Ramón y Cajal de Investigaciones Sanitarias (IRYCIS), Madrid, Spain

## Abstract

Crc is a post-transcriptional regulator in *Pseudomonas aeruginosa* that modulates its metabolism, but also its susceptibility to antibiotics and virulence. Most of *P. aeruginosa* virulence factors are secreted or engulfed in vesicles. A Crc deficient mutant was created and the extracellular vesicles associated exoproteome and the vesicle-free secretome was quantified using iTRAQ. Fifty vesicles-associated proteins were more abundant and 14 less abundant in the Crc-defective strain, whereas 37 were more abundant and 17 less abundant in the vesicle-free secretome. Different virulence determinants, such as ToxA, protease IV, azurin, chitin-binding protein, PlcB and Hcp1, were less abundant in the Crc-defective mutant. We also observed that the crc mutant presented an impaired vesicle-associated secretion of quorum sensing signal molecules and less cytotoxicity than its wild-type strain, in agreement with the low secretion of proteins related to virulence. Our results offer new insights into the mechanisms by which Crc regulates *P. aeruginosa* virulence, through the modulation of vesicle formation and secretion of both virulence determinants and quorum sensing signals.

Specifications table

**Table:** 

Subject area	Biology
More specific subject area	Microbiology
Type of data	Tables
How data was acquired	Proteomic analysis of extracellular vesicles (EVs) and vesicle-free secretome of Pseudomonas aeruginosa PAO1 and FCP001 (Crc-defective strain)
Data format	Analyzed
Experimental factors	Secretome extraction, extracellular vesicles isolation and vesicle-free secretome concentration
Experimental features	Extracellular vesicles and vesicle free secretome were obtained from Pseudomonas aeruginosa PAO1 (wild-type strain) and FCP001 (crc-defective mutant), proteins were solubilized and digested. Peptides were labeled with iTRAQ reagents. Proteins were identified and quantified by nano-LC ESI-MSMS
Data source location	Madrid, Spain
Data accessibility	Data are available with this paper

Value of the data

• The exoproteome of *P. aeruginosa* is analyzed using iTRAQ technology
• The vesicle-associated proteome of *P. aeruginosa* is analyzed using iTRAQ technology.
• Crc modulates the secretion of virulence factors and generation of vesicles in *P. aeruginosa*.

## 1 Data, experimental design, materials and methods

The data show the lists of proteins identified and quantified in Extracellular Vesicles (EVs) and in the vesicle-free secretome fraction of the wild-type strain *Pseudomonas aeruginosa* PAO1 and its isogenic *crc*-deffective derivative FCP001. The analysis was performed by nano-LC ESI-MSMS analysis using a nano-liquid chromatography system coupled to high speed Triple TOF 5600 mass spectrometer with a duo spray ionization source. The proteins were quantified and processed using Analyst^®^ TF 1.5.1 Software (AB SCIEX). Table 1 contains a list of 1058 proteins identified in the *P. aeruginosa* vesicle-free secretome and Table 2 contains 643 quantified proteins with 2 or more peptides in the same fraction. Table 3 contains a list of 839 proteins identified in the *P. aeruginosa* EVs fraction. Table 4 list the 489 proteins with 2 or more peptides identified in EVs sample. Of these, 37 proteins were more abundant and 16 were less abundant in the vesicle-free secretome and 50 proteins were more abundant and 14 less abundant in the EVs of the FCP001 mutant defective in *crc* than they were in the control PAO001 parental strain [1]. All mass spectrometry proteomics data have been deposited to the ProteomeXchange Consortium (http://proteomecentral.proteomexchange.org) via the PRIDE partner repository [2] using MIAPE Extractor v3.7.2, with the dataset identifier PXD000687 and DOI 10.6019/PXD000687.

## 2 Isolation of extracellular vesicles and vesicle-free secretome

Three biological replicates of *P. aeruginosa* PAO001 and FCP001 were obtained from mid-exponential growth phase cultures (OD600 of 0.6). Extracellular vesicles were concentrated using a Centricon Plus-70 filter (>100 kDa) and pelleted by ultracentrifuged at 100,000×*g* for 1 h at 4 °C. The supernatants were collected, mixed with the flowthrough <100 kDa and concentrated using a 10 kDa cut-off filter to obtain the vesicle-free secretome. Proteins were solubilised in 0.5 M Triethylammonium bicarbonate (TEAB) buffer supplemented with a protease inhibitor cocktail.

## 3 Quantitative proteomic analysis

### 3.1 Sample preparation

Vesicle and vesicle-free secretomes were treated and analyzed independently. Samples were precipitated with methanol/chloroform and resuspended in 0.5 M Triethylammonium bicarbonate (TEAB). 30 µg of protein from each condition were used for the trypsin digestion. Proteins were denatured in 6 M guanidine hydrochloride/100 mM HEPES, pH 7.5, reduced in 50 mM Tris (2-carboxyethyl) phosphine (TCEP, AB SCIEX), pH 8.0, at 60 °C for 60 min, and 200 mM cysteine-blocking reagent (methyl methanethiosulfonate (MMTS, Pierce) were added for 10 min at room temperature. Samples were digested by adding 3 µl (1 µg/µl) sequence grade-modified trypsin to each sample in a ratio 1/10 (w/w). The samples were then incubated at 37 °C overnight on a shaker and evaporated to dryness.

### 3.2 iTRAQ labeling

Secretome and vesicle-free secretome digested samples were labeled at room temperature for 2 h with a half-unit of iTRAQ Reagent Multi-plex kit (AB SCIEX, Foster City, CA, USA) previously reconstituted with 80 μl of 70% ethanol/50 mM TEAB. The iTRAQ labeling was performed separately in a 2-plex design for each condition. In the first labeling (iTRAQ1), tags 114 and 115 were used for the vesicle-free secretome samples. In the second labeling (iTRAQ2), tags 116 and 117 were used for vesicle samples. After that, samples were combined and labeling reaction was stopped by adding 100 µl of 50% ACN, followed by evaporation of the samples in a vacuum concentrator.

The digested, labeled and pooled peptide mixtures were desalted using a Sep-PAK C18 Cartridge (Waters), following manufacture indications; the cleaned tryptic peptides were evaporated to dryness and stored at −20 °C for further analysis.

### 3.3 Liquid chromatography and mass spectrometer analysis

A 2.5 µg aliquot of each peptide mixture was subjected to 2D-nano-LC ESI-MSMS analysis using a nano-liquid chromatography system coupled to high speed Triple TOF 5600 mass spectrometer with a duo spray ionization source. The analytical column used was a silica-based reversed phase column C18 ChromXP 75 µm×15 cm, 3 µm particle size and 120 Å pore. The trap column was a C18 ChromXP, 3 µm particle diameter, 120 Å pore size, switched on-line with the analytical column. The loading pump delivered a solution of 0.1% formic acid in water at 2 µl/min. The nano-pump provided a flow-rate of 300 nl/min and was operated under gradient elution conditions, using 0.1% formic acid in water as mobile phase A, and 0.1% formic acid in acetonitrile as mobile phase B. Gradient elution was performed according the following scheme: isocratic conditions of 98% A: 2% B for 1 min, a linear increase to 30% B in 120 min, a linear increase to 40% B in 10 min, a linear increase to 90% B in 5 min, isocratic conditions of 90% B for 5 min and return to initial conditions in 2 min. Injection volume was 5 µl.

Data acquisition was performed with a Triple TOF 5600 System. Data was acquired using an ionspray voltage floating (ISVF) 2800 V, curtain gas (CUR) 20, interface heater temperature (IHT) 150, ion source gas 1 (GS1) 20, declustering potential (DP) 85 V. All data were acquired using information-dependent acquisition (IDA) mode with Analyst TF 1.5 software. For IDA parameters, 0.25 s MS survey scan in the mass range of 350–1250 Da was followed by 15 MS/MS scans of 250 ms in the mass range of 100–1800 (total cycle time: 4.04 s). Switching criteria were set to ions greater than mass to charge ratio (*m*/*z*) 350 and smaller than *m*/*z* 1250 with charge state of 2–5 and an abundance threshold of more than 90 counts (cps). Former target ions were excluded for 20 s. IDA rolling collision energy (CE) parameters script was used for automatically controlling the CE.

All mass spectrometry proteomics data have been deposited to the ProteomeXchange Consortium (http://proteomecentral.proteomexchange.org) via the PRIDE partner repository [2] using MIAPE Extractor v3.7.2, with the dataset identifier PXD000687 and DOI 10.6019/PXD000687.

### 3.4 Data analysis

MS and MS/MS data obtained for pooled samples were processed using Analyst^®^ TF 1.5.1 Software. Raw data file conversion tools generated mgf files which were also searched against the *P. aeruginosa* (PAO001) database, containing 5563 protein sequences and their corresponding reversed entries using the Mascot Server v. 2.3.02. Search parameters were set as follows: enzyme, trypsin; allowed missed cleavages, 1; fixed modifications, iTRAQ 4-plex (N-term and K) and beta-methylthiolation of cysteine; variable modifications, oxidation of methionine. Peptide mass tolerance was set to ±25 ppm for precursors and 0.05 Da for fragment masses. Frequency distribution histograms of protein ratios were obtained into Excel 2010. Log_2_ peptide ratios followed a normal distribution that was fitted using least squares regression. Mean and standard deviation values derived from the Gaussian fit were used to calculate *p*-values and quantification of False Discovery Rates (FDR).The confidence interval for protein identification was set to ≥95% (*p*<0.05) and only peptides with an individual ion score above the 1% False Discovery Rates (FDR) threshold were considered correctly identified. Only proteins having at least two quantitated peptides were considered in the quantitation. Changes among the different samples were considered as relevant when the ratio between PAO001 and FCP001 were ±1 (Log_2_) in, at least two of the three biological replicates.
